# Numerical Investigation and Device Architecture Optimization of Sb_2_Se_3_ Thin-Film Solar Cells Using SCAPS-1D

**DOI:** 10.3390/ma17246203

**Published:** 2024-12-19

**Authors:** Chung-Kuan Lai, Yi-Cheng Lin

**Affiliations:** Department of Mechatronics Engineering, National Changhua University of Education, Changhua 50007, Taiwan; cklai0703@gmail.com

**Keywords:** Sb_2_Se_3_ solar cells, hole-transport layer, SCAPS-1D, shallow acceptor density, series and shunt resistance

## Abstract

Antimony selenide (Sb_2_Se_3_) shows promise for photovoltaics due to its favorable properties and low toxicity. However, current Sb_2_Se_3_ solar cells exhibit efficiencies significantly below their theoretical limits, primarily due to interface recombination and non-optimal device architectures. This study presents a comprehensive numerical investigation of Sb_2_Se_3_ thin-film solar cells using SCAPS-1D simulation software, focusing on device architecture optimization and interface engineering. We systematically analyzed device configurations (substrate and superstrate), hole-transport layer (HTL) materials (including NiOx, CZTS, Cu_2_O, CuO, CuI, CuSCN, CZ-TA, and Spiro-OMeTAD), layer thicknesses, carrier densities, and resistance effects. The substrate configuration with molybdenum back contact demonstrated superior performance compared with the superstrate design, primarily due to favorable energy band alignment at the Mo/Sb_2_Se_3_ interface. Among the investigated HTL materials, Cu_2_O exhibited optimal performance with minimal valence-band offset, achieving maximum efficiency at 0.06 μm thickness. Device optimization revealed critical parameters: series resistance should be minimized to 0–5 Ω-cm^2^ while maintaining shunt resistance above 2000 Ω-cm^2^. The optimized Mo/Cu_2_O(0.06 μm)/Sb_2_Se_3_/CdS/i-ZnO/ITO/Al structure achieved a remarkable power conversion efficiency (PCE) of 21.68%, representing a significant improvement from 14.23% in conventional cells without HTL. This study provides crucial insights for the practical development of high-efficiency Sb_2_Se_3_ solar cells, demonstrating the significant impact of device architecture optimization and interface engineering on overall performance.

## 1. Introduction

Antimony selenide (Sb_2_Se_3_) represents a promising advancement in photovoltaic technology, characterized by its unique one-dimensional crystal structure and tunable direct bandgap (1.2–1.9 eV) [1]. This material system offers multiple advantages for solar-cell applications, including exceptional photoelectric properties, high absorption coefficient, minimal environmental impact, and cost-effectiveness due to earth-abundant constituents [2]. However, Sb_2_Se_3_ solar cells face significant performance limitations, primarily due to reduced open-circuit voltage resulting from carrier recombination at metal back contact interfaces and performance degradation caused by surface oxidation [3]. Various strategies have been implemented to enhance Sb_2_Se_3_ solar-cell performance, including doping mechanisms, defect engineering, and interface optimization [4,5]. Notable advances include tellurium doping for defect passivation and transition metal oxide implementation (NiO_x_, MoO_x_) for band alignment optimization. Recent developments, such as the solvent-assisted hydrothermal deposition (SHD) technique, have achieved efficiency improvements of up to 10.75% [6]. Nevertheless, these efficiencies remain significantly below the theoretical Shockley–Queisser (S-Q) limit and the performance of conventional thin-film photovoltaics.

The HTL plays a crucial role in thin-film solar cells by facilitating efficient hole transport while blocking electrons, thereby preventing charge recombination. Additionally, it optimizes energy-level alignment between the active layer and electrode while enhancing interface quality, ultimately improving PCE [7]. Contemporary research encompasses various HTL materials, including NiO_x_ [8,9], CZTS [10,11], CuI [12], Cu_x_O [13,14], CuSCN [15,16], CZ-TA [17,18], AgInTe_2_ [19], MoSe_2_ [20,21], and Spiro-OMeTAD [22,23,24]. While experimental studies dominate the field, comprehensive numerical investigations incorporating device structure optimization with HTL material selection remain limited. Among available solar-cell simulation tools, including AMPS1D and wxAMPS-1D [25,26,27], SCAPS-1D [12,17,19,28,29,30] has emerged as the preferred platform for Sb_2_Se_3_ solar-cell research. This study employed SCAPS-1D software (version 3.3.11) to conduct comprehensive numerical simulation and performance optimization of Sb_2_Se_3_ thin-film solar cells. The investigation encompassed device structures (substrate and superstrate configurations), HTL material optimization, thickness effects, shallow acceptor densities, and the impact of series and shunt resistance parameters. The aim of these simulations was to accelerate theoretical understanding and facilitate performance breakthroughs in Sb_2_Se_3_ solar-cell technology.

## 2. Device Structure and Simulation Parameters

### 2.1. Device Structure

**Figure 1:**
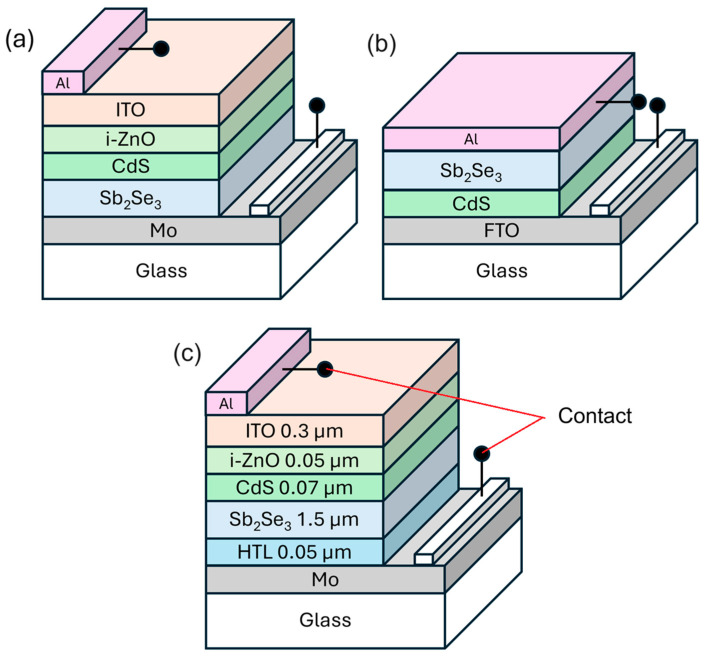
Schematic diagram of the proposed solar-cell structure: (**a**) p-n substrate configuration, (**b**) p-n superstrate configuration, (**c**) n-p-p^+^ substrate configuration.

Solar-cell device structures can be classified into two main categories: substrate and superstrate configurations. Figure 1a, with CdS positioned below the absorber layer, represents the substrate configuration [31], while Figure 1b, with CdS located above the absorber layer, illustrates the superstrate configuration. This investigation systematically examines three distinct device architectures: substrate and superstrate configurations without HTLs (Figure 1a and Figure 1b, respectively), followed by an advanced architecture incorporating an HTL (Figure 1c). In these solar-cell structures, molybdenum (Mo), fluorine-doped tin oxide (FTO), and aluminum (Al) serve as electron-collecting front contacts and hole-collecting back contacts. Antimony selenide (Sb_2_Se_3_) functions as the primary absorber layer, while cadmium sulfide (CdS) serves as the wide-bandgap window layer. Adjacent to the buffer layer, a highly resistive intrinsic zinc oxide (i-ZnO) layer is implemented, overlaid with tin-doped indium oxide (ITO) functioning as a transparent conductive oxide (TCO) to facilitate efficient charge collection and transport through the device. Additionally, i-ZnO acts as a buffer layer between CdS and ITO layers, while its undoped nature reduces interface recombination. It aids in band alignment for efficient charge transport and protects underlying layers during ITO deposition.

### 2.2. Numerical Method

The analysis of SCAPS-1D (version 3.3.11) is based upon Poisson’s equation, hole continuity, and electron continuity, as given below [29]:

(1) $$\frac{\partial^{2}\varphi }{\partial x^{2}}+\frac{q}{\varepsilon }\left[p\left(x\right)-n\left(x\right)+N_{D}-N_{A}+\rho_{p}-\rho_{n}\right]=0$$

(2) $$\frac{1}{q}\frac{dJ_{p}}{d_{x}}=G_{op}\left(x\right)-R(x)$$

(3) $$\frac{1}{q}\frac{dJ_{n}}{d_{x}}={-G}_{op}\left(x\right)-R\left(x\right)$$

where *ε* is the dielectric constant; *q* is the electron charge; *N_A_* and *N_D_* are acceptor and donor type density, respectively; *φ* is the electrostatic potential; and *p*, *n*, *ρ_p_*, *ρ_n_*, *J_p_*, and *J_n_* are hole concentration, electron concentration, hole distribution, electron distribution, current densities of holes, and current densities of electrons, respectively. *G_op_* is the optical generation rate, and *R* is the net recombination from direct and indirect recombination. All of these parameters are the function of the position coordinate *x*.

The numerical simulation implemented in SCAPS-1D software requires comprehensive material parameters for each layer of the device structure. All material properties and parameters utilized in this study are derived from established literature [8,10,32,33,34,35,36,37,38,39]. The simulation parameters are systematically organized in Table 1, Table 2, Table 3 and Table 4, encompassing fundamental material properties of the Sb_2_Se_3_ device, interface defect characteristics, HTL material specifications, and electrode parameters. Key simulation parameters include electron and hole capture cross-sections for both bulk and interface defects (10^−15^ cm^2^), radiative recombination coefficient (10^−8^ cm/s), and carrier thermal velocities (10^7^ cm/s for both electrons and holes, as detailed in Table 3). All simulations were performed under standardized conditions of 300 K and AM 1.5 G illumination (100 mW/cm^2^), with initial analyses conducted without considering series and shunt resistance effects.

## 3. Results and Discussion

### 3.1. Comparison of Substrate and Superstrate Configurations

**Figure 2:**
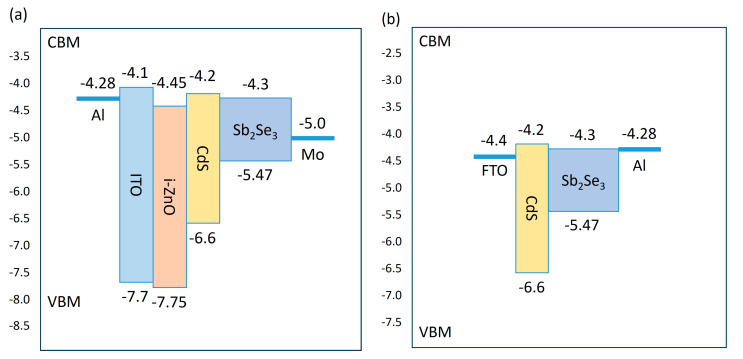
Energy band diagrams of different device configurations: (**a**) substrate and (**b**) superstrate structures.

**Figure 3:**
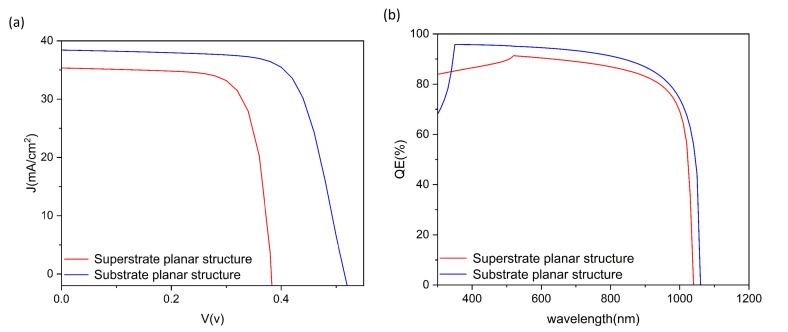
Performance characteristics of different device configurations: (**a**) current–voltage curves and (**b**) external quantum efficiency spectra.

This investigation presents a systematic comparison of substrate and superstrate architectures in Sb_2_Se_3_ solar cells. The substrate configuration implements a Mo/Sb_2_Se_3_/CdS/i-ZnO/ITO/Al structure (Figure 1a), whereas the superstrate variant employs a FTO/CdS/Sb_2_Se_3_/Al architecture (Figure 1b). Energy band alignments for both configurations are depicted in Figure 2. To ensure comparative validity, material layers and metal electrodes remained consistent, with contact parameters specified in Table 4. Utilizing parameters detailed in Table 1 and Table 2, we obtained current–voltage characteristics and external quantum efficiency (EQE) curves through numerical simulation (Figure 3), with performance metrics compiled in Table 5.

Energy band structure analysis (Figure 2) reveals that the primary differentiation between configurations manifests in the electrode/absorber interface characteristics. The substrate configuration exhibits favorable energetics, with minimal energy-level difference between the Mo electrode (work function 4.9 eV) and Sb_2_Se_3_ valence band (5.5 eV), enabling formation of a quasi-ohmic contact. This advantageous band alignment substantially reduces interfacial charge transfer resistance and minimizes non-radiative recombination at interface states. Conversely, the superstrate configuration’s Al electrode, characterized by a lower work function (4.28 eV), generates a significant Schottky barrier (approximately 1.22 eV), hindering efficient photogenerated hole collection. Substitution of Al with Au (work function 5.1 eV) as the back contact reduces barrier height to approximately 0.4 eV, substantially improving interfacial characteristics. This enhancement manifests in device parameters (Table 5), elevating both the fill factor (FF) and open-circuit voltage (Voc) through reduced interface recombination.

Current–voltage analysis (Figure 3a) demonstrates the substrate configuration’s superior performance, achieving higher open-circuit voltage (0.52 V) and short-circuit current density (38.42 mA/cm^2^) compared with the superstrate design (Voc = 0.38 V, Jsc = 35.34 mA/cm^2^). This performance enhancement primarily originates from the quasi-ohmic contact at the Mo/Sb_2_Se_3_ interface, where optimal band alignment minimizes carrier injection/extraction barriers and voltage losses. EQE measurements (Figure 3b) further validate this advantage, with the substrate configuration exhibiting enhanced photoresponse across 400–1000 nm, particularly maintaining high quantum efficiency in the long-wavelength region (>800 nm). These characteristics indicate superior minority carrier collection efficiency and reduced bulk recombination. Despite a slightly lower fill factor in the substrate configuration (71.88% vs. 74.62%), potential exists for enhancement through interface engineering.

Significantly, implementation of Au electrodes in the superstrate configuration elevates performance (PCE = 13.83%) near that of the substrate configuration (PCE = 14.23%), confirming the critical role of electrode/absorber interface band alignment. Based on comprehensive evaluation of performance metrics, process compatibility, and interface stability, we selected the substrate configuration for subsequent optimization studies. This architecture demonstrates both superior photovoltaic conversion efficiency potential and clear pathways for further performance enhancement in Sb_2_Se_3_ solar cells.

### 3.2. Comparison of Different HTL Materials

**Figure 4:**
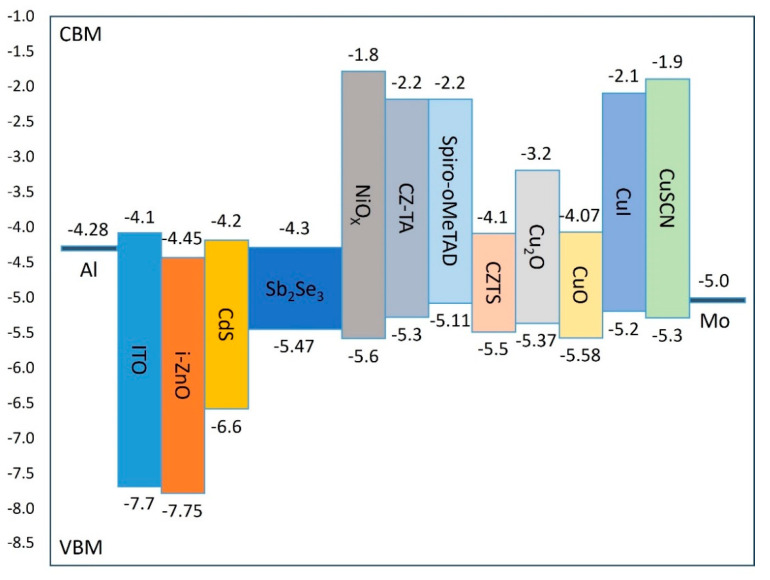
Energy band diagrams of various HTL materials in the device structure.

This investigation examines the performance characteristics of various HTL materials, with simulation parameters documented in Table 3. Figure 4 illustrates the energy band diagrams of different HTL materials within the complete device structure. Two critical band alignment requirements govern HTL material selection: First, the valence-band maximum (VBM) of the HTL must be positioned appropriately relative to both the back contact work function and the Sb_2_Se_3_ VBM to facilitate efficient hole transfer. As is evident from the band diagrams, the VBMs of NiOx, CuO, and CZTS lie slightly below that of the Sb_2_Se_3_ absorber layer, creating potential barriers that impede effective hole transport. Second, the conduction band minimum (CBM) of the HTL must exceed that of Sb_2_Se_3_ to prevent electron backflow. Although CuO and CZTS exhibit CBM levels marginally higher than the Sb_2_Se_3_ absorber layer, their relatively small energy offsets with the buffer layer results in inadequate electron blocking, failing to effectively prevent electron drift towards the back contact and increasing carrier recombination probability.

**Figure 5:**
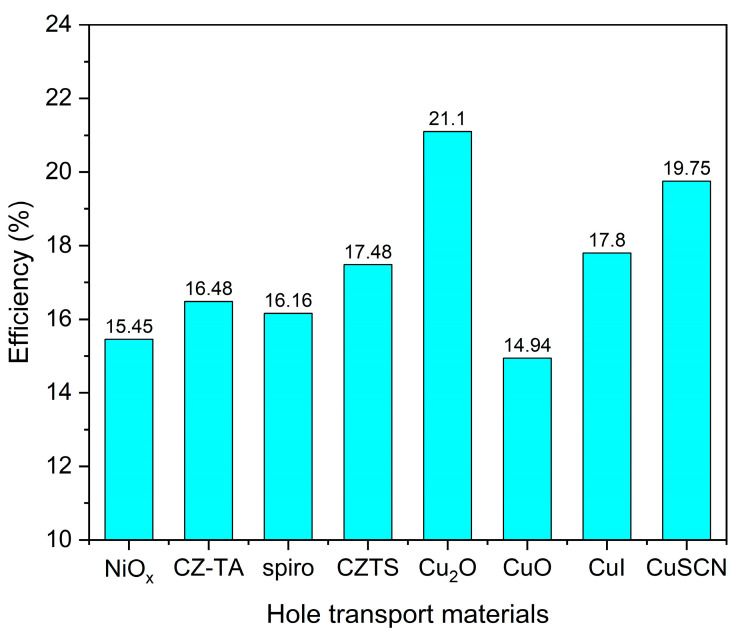
PCE comparison of different HTL materials.

Table 6 presents the valence-band offset (VBO) values between each HTL and the Sb_2_Se_3_ absorber layer, calculated using the modified Equation (2). These VBO values significantly impact device performance: positive VBO values (such as Cu_2_O at 0.1 eV, CZ-TA at 0.17 eV, and Spiro at 0.36 eV) create appropriate energy barriers at the absorber/HTL interface, contributing to electron blocking functionality. Conversely, negative VBO values (such as NiOx at −0.13 eV and CuO at −0.11 eV) form energy wells at the interface, increasing carrier recombination probability. Simulation results, as shown in Figure 5, demonstrate that Cu_2_O achieves the highest PCE (21.1%) as an HTL material due to its moderate positive VBO (0.1 eV), which provides electron blocking capabilities without excessively hindering hole transport. This optimal performance is further enhanced by Cu_2_O’s superior carrier mobility characteristics.

Analysis reveals several key structure−property relationships. Materials with moderate positive VBO values (0.1–0.2 eV) exhibit superior performance by achieving an optimal balance between electron blocking and hole transport. Cu_2_O’s exceptional performance stems from its optimal band alignment and high carrier mobility (approximately 10^−2^ cm^2^/V·s), facilitating efficient hole extraction. In contrast, materials with larger positive VBO values, such as Spiro (0.36 eV), generate excessive barriers for hole transport despite effective electron blocking. The negative VBO values observed in NiOx and CuO not only fail to block electrons effectively but also create potential wells that trap holes, leading to increased interface recombination and reduced device efficiency.

### 3.3. Effects of Cu_2_O Thickness and Shallow Acceptor Density on Device Performance

**Figure 6:**
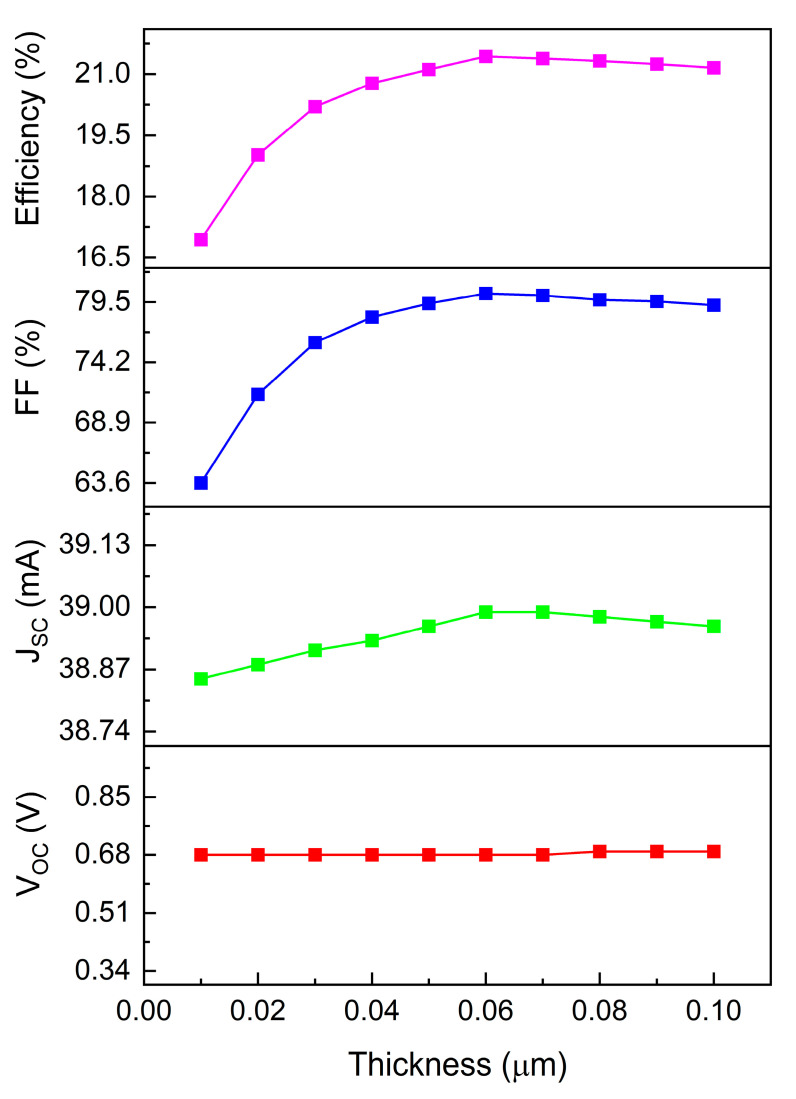
Relationship between Cu_2_O HTL thickness and device performance.

The influence of Cu_2_O thickness on device performance is characterized in Figure 6, with thickness variations examined from 0.01 μm to 0.1 μm. Throughout this range, the open-circuit voltage (Voc) maintains relative stability at approximately 0.68 V, attributable to ideal solar cell behavior where light penetration and minority carrier collection remain optimal. Increasing Cu_2_O thickness extends the optical path length through the HTL, enhancing photon absorption and carrier generation, consequently improving short-circuit current density (Jsc). An optimized HTL thickness facilitates balanced electron and hole transport rates, minimizing interface charge accumulation and enhancing the fill factor. However, excessive Cu_2_O layer thickness leads to carrier recombination before electrode collection, reducing carrier collection efficiency, conductivity, Jsc, fill factor, and overall performance. Simulation results establish optimal photoelectric conversion efficiency of 21.43% at 0.06 μm thickness. Shallow acceptor density characterizes the concentration of hole acceptors proximate to the conduction band edge in semiconductors. These acceptors, introduced through impurity atoms or intrinsic defects, exhibit energy levels within 0.1 eV of the conduction band edge, effectively capturing photo-excited holes and reducing recombination losses, thereby enhancing photoelectric conversion efficiency.

**Figure 7:**
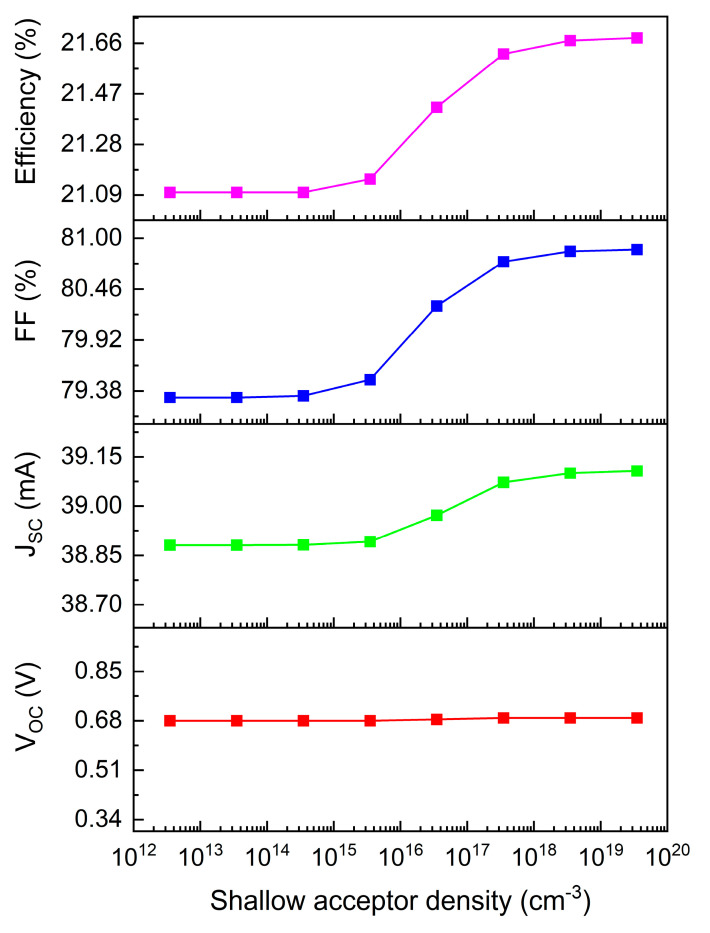
Relationship between Cu_2_O HTL shallow acceptor density and device performance.

Figure 7 illustrates the correlation between Cu_2_O HTL shallow acceptor density and device performance. Performance enhancement becomes significant above 10^16^ cm^−3^, corresponding to the absorption layer’s shallow acceptor density. Exceeding this threshold creates an effective back potential barrier, with increased shallow acceptor density improving conductivity, Jsc, fill factor, and overall efficiency while reducing series resistance. However, continued density increases shift the Fermi level toward the valence band. At excessive densities, the Fermi level enters the valence band, creating high hole concentrations and transforming the semiconductor into a strong p-type conductor, potentially causing simulation convergence failures.

**Figure 8:**
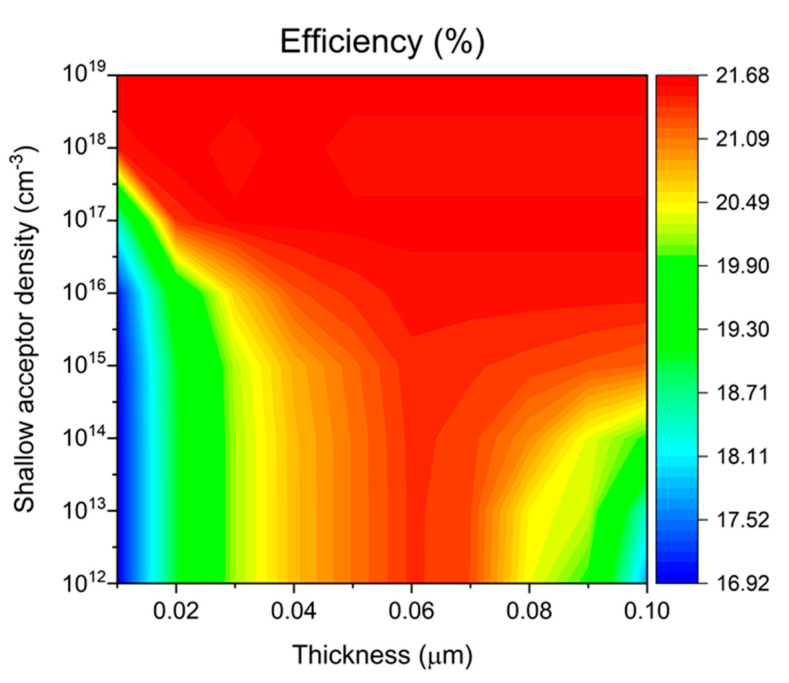
Effect of shallow acceptor density on Sb_2_Se_3_ solar-cell efficiency at different Cu_2_O HTL thicknesses.

Figure 8 presents a comprehensive visualization of device efficiency as a function of both Cu_2_O HTL thickness and shallow acceptor density, displayed through a color-mapped surface plot. In the low acceptor density region (10^12^–10^15^ cm^−3^), device efficiency remains relatively low, particularly when combined with HTL thicknesses below 0.04 μm. The plot reveals a critical transition around 10^16^ cm^−3^ acceptor density, above which device performance significantly improves. Notably, devices with HTL thickness near 0.06 μm demonstrate optimal performance and exhibit greater stability across acceptor density variations, suggesting a favorable processing window for device fabrication. The highest efficiency region, indicated by the red area in the plot, occurs at the combination of 0.06 μm HTL thickness and acceptor densities above 10^18^ cm^−3^. Under these optimal conditions (0.06 μm thickness, 10^19^ cm^−3^ acceptor density), the device achieves peak performance parameters: Voc = 0.69 V, Jsc = 39.11 mA/cm^2^, FF = 80.88%, and overall efficiency of 21.68%. This efficiency enhancement can be attributed to improved carrier transport and reduced interface recombination at higher acceptor densities, while the optimal thickness ensures effective light absorption without excessive carrier transport losses.

### 3.4. Effects of Series and Shunt Resistance on Device Performance

**Figure 9:**
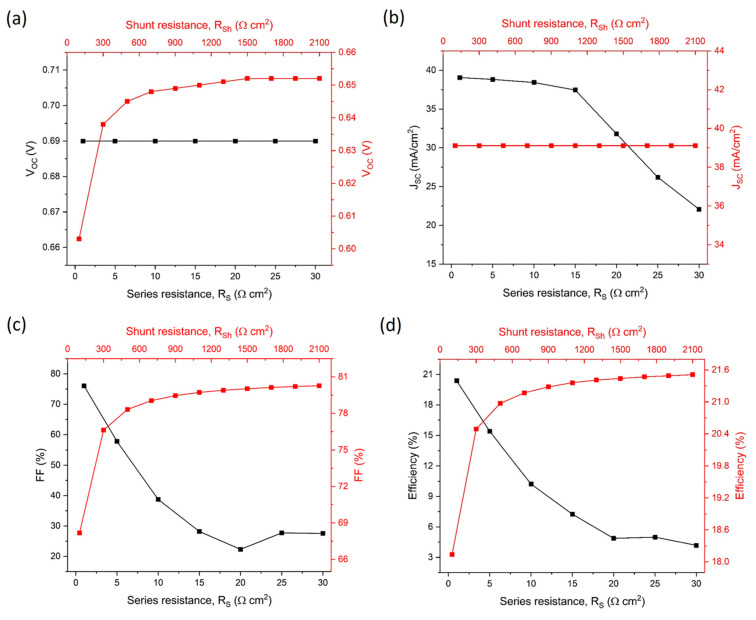
Numerical analysis of series and parallel resistance on device performance. (**a**) Open-circuit voltage (Voc) variation, (**b**) Short-circuit current density (Jsc) response, (**c**) Fill Factor (FF) dependence, and (**d**) Device efficiency changes with respect to series and parallel resistance.

To investigate the influence of series resistance (Rs) and shunt resistance (Rsh), we maintained all other parameters at their optimized values. The impact of these resistances on solar-cell performance is illustrated in Figure 9, with Rs varying from 1–30 Ω-cm^2^ and Rsh from 100–2100 Ω-cm^2^. Both parameters demonstrate significant influence on overall device efficiency. Figure 9a,b reveal distinct relationships between resistance parameters and device characteristics: the open-circuit voltage (Voc) exhibits slight enhancement with increasing Rsh while remaining independent of Rs variations. Conversely, short-circuit current density (Jsc) shows strong Rs dependence while maintaining relative stability with Rsh variations. This behavior aligns with Ohm’s law (V = IR): during open-circuit conditions, current absence through Rs results in zero voltage drop, rendering Voc independent of Rs magnitude. Under short-circuit conditions, the external circuit resistance becomes negligible compared to Rsh, directing photogenerated current predominantly through the external circuit. Analysis of Figure 9c,d reveals substantial performance dependencies: increasing Rs severely degrades the fill factor (FF) from 76.05% to 27.55%, with corresponding efficiency reduction from 20.38% to 4.18%. In contrast, enhanced Rsh improves device performance, elevating FF from 68.18% to 80.27% and efficiency from 18.14% to 21.51%. These findings establish optimal performance parameters for Sb_2_Se_3_ solar cells: Rs should be minimized to 0–5 Ω-cm^2^, while Rsh should exceed 2000 Ω-cm^2^.

## 4. Conclusions

This study utilized SCAPS-1D software to conduct numerical simulation and performance optimization of Sb_2_Se_3_ thin-film solar cells. The investigation focused on key parameters including solar-cell device structures (substrate and superstrate configurations), various hole-transport layer (HTL) materials (NiO_x_, CZTS, Cu_2_O, CuO, CuI, CuSCN, CZ-TA, and Spiro-OMeTAD), their thicknesses, shallow acceptor densities, and the effects of series and shunt resistances. Through systematic analysis, the substrate configuration with Mo back contact demonstrated superior performance compared to the superstrate configuration. Among the various HTL materials investigated, Cu_2_O exhibited the highest photoelectric efficiency due to its smallest VBO value. The study revealed that HTL thickness control is more critical than carrier concentration control, with Cu_2_O HTL achieving optimal photoelectric conversion efficiency at 0.06 μm thickness. This configuration provided a wide process window, as the efficiency showed relatively low sensitivity to changes in shallow acceptor density. For achieving high-efficiency Sb_2_Se_3_ solar cells, the research established that series resistance should be reduced (to 0–5 Ω-cm^2^) and shunt resistance increased (to above 2000 Ω-cm^2^), which significantly improved both the fill factor and photoelectric conversion efficiency. Under optimized conditions utilizing the Mo/Cu_2_O(0.06 μm)/Sb_2_Se_3_/CdS/i-ZnO/ITO/Al structure, the device showed significantly improved photoelectric conversion efficiency from 14.23% (conventional cells without HTL) to a maximum of 21.68% with the optimized Cu_2_O HTL.

Looking forward, several promising research directions emerge from this work. Further interface engineering studies could focus on novel buffer layer materials and surface passivation techniques to minimize recombination losses. Material development efforts should explore new HTL alternatives with enhanced band alignment and carrier transport properties, while also considering stability and cost-effectiveness. Device architecture optimization could investigate tandem configurations and alternative contact materials. Additionally, practical implementation aspects such as scalable fabrication processes, long-term stability, and cost–benefit analyses of different materials and structures warrant investigation. These future directions are aimed at bridging the gap between simulated theoretical performance and practical device implementation, ultimately advancing the development of high-efficiency Sb_2_Se_3_ solar cells for real-world applications.

## Figures and Tables

**Table 1:** Materials parameters used in the simulation.

Parameter	ITO [32]	i-ZnO [33]	CdS [33]	Sb_2_Se_3_ [33]
Thickness (µm)	0.3	0.05	0.07	1.5
E_g_ (eV)	3.6	3.3	2.4	1.17
χ (eV)	4.1	4.45	4.2	4.3
ε_r_	10	9	10	19
N_C_ (1 cm^−3^)	2.2 × 10^18^	2.2 × 10^18^	2.2 × 10^18^	2.2 × 10^18^
N_V_ (1 cm^−3^)	1.8 × 10^19^	1.8 × 10^19^	1.8 × 10^19^	1.8 × 10^19^
υ_th,e_ (cm/s)	10^7^	10^7^	10^7^	10^7^
υ_th,h_ (cm/s)	10^7^	10^7^	10^7^	10^7^
μ_e_ (cm^2^ (V S)^−1^)	75	100	100	15
μ_h_ (cm^2^ (V S)^−1^)	50	25	25	42
Donor density, N_D_ (1/cm^3^)	10^19^	10^18^	10^17^	0
Acceptor density, N_A_ (1 cm^−3^)	0	10^18^	10^2^	10^16^
Defect type		Acceptor	Donor	Neutral
Reference		Above E_V_	Above E_V_	Above E_V_
E_t_ (eV)		0.6	0.6	0.6
N_t_ (1 cm^−3^)		10^10^	10^15^	10^12^

χ: electron affinity, ε_r_: dielectric permittivity (relative), N: effective density of states, C: conduction band, V: valence band, υ_th_: thermal velocity, μ: mobility, N_A_: shallow acceptor density, N_D_: shallow donor density, E_t_: trap position, N_t_: trap density.

**Table 2:** Interface defect simulation parameters used in the simulation.

Interface	i-ZnO/CdS [33]	CdS/Sb_2_Se_3_ [33]	Sb_2_Se_3_/HTL [34]
Defect type	Neutral	Neutral	Neutral
σ_e_ (cm^2^)	4 × 10^−18^	1 × 10^−19^	1 × 10^−19^
σ_h_ (cm^2^)	4 × 10^−18^	1 × 10^−19^	1 × 10^−19^
N_t_ (1 cm^−2^)	10^10^	2.8 × 10^10^	10^12^

σ: capture cross-section, N_t_: trap density.

**Table 3:** Simulation parameters of HTL materials used in the simulation.

Parameter	CZ-TA [17]	Spiro [17]	CZTS [11]	Cu_2_O [35]	CuO [35]	CuI [35]	CuSCN [36]	NiO_x_ [36]
Thickness (µm)	0.05	0.05	0.05	0.05	0.05	0.05	0.05	0.05
E_g_ (eV)	3.10	2.91	1.4	2.17	1.51	3.1	3.4	3.8
χ (eV)	2.2	2.2	4.1	3.2	4.07	2.1	1.9	1.8
ε_r_	3	3	9	7.11	18.1	6.5	10.0	11.75
N_C_ (cm^−3^)	8 × 10^17^	8 × 10^17^	2.2 ×10^18^	2.02 × 10^17^	2.2 × 10^19^	2.8 × 10^19^	2.2 × 10^18^	2 × 10^18^
N_V_ (cm^−3^)	1.8 × 10^19^	1.8 × 10^19^	1.8 × 10^18^	1.1 × 10^19^	5.5 × 10^20^	1.0 × 10^19^	1.8 × 10^18^	2 × 10^18^
μ_e_ (cm^2^ (VS)^−1^)	1.65 × 10^−4^	6.17 × 10^−5^	100	200	10	100	100	8
μ_h_ (cm^2^ (VS) ^−1^)	1.65 × 10^−4^	6.17 × 10^−5^	12.5	80	0.1	43.9	25	81.10
N_D_ (1 cm^−3^)	0	0	0	0	0	0	0	0
N_A_ (1 cm^−3^)	10^19^	10^19^	10^19^	10^19^	10^19^	10^19^	10^19^	10^19^

**Table 4:** Simulation parameters of contact materials used in the simulation.

Contacts	Al [37]	Mo [38]	FTO [39]
W_f_	4.28	5.0	4.4
S_e_	10^7^	10^5^	10^7^
S_h_	10^5^	10^7^	10^5^
Reflection	No	No	No

W_f_: work function, S: surface recombination velocity.

**Table 5:** Device performance parameters for different configuration structures.

	V_OC_ (V)	$J_{\mathrm{SC}}\;(mA/{cm}^{2})$	FF (%)	PCE (%)
Substrate	0.52	38.42	71.88	14.23
Superstrate	0.38	35.34	74.62	10.09
Superstrate with Au (W_f_ = 5.1)	0.52	35.66	75.21	13.83

**Table 6:** Calculated VBO values for different HTL materials.

HTL	NiO_X_	CZ-TA	Spiro	CZTS	Cu_2_O	CuO	CuI	CuSCN
VBO	−0.13	0.17	0.36	−0.03	0.1	−0.11	0.27	0.17

## Data Availability

The original contributions presented in this study are included in the article. Further inquiries can be directed to the corresponding author.
